# Childhood development of brain white matter myelin: a longitudinal T1w/T2w-ratio study

**DOI:** 10.1007/s00429-023-02718-8

**Published:** 2023-11-20

**Authors:** Lillian M. Dipnall, Joseph Y. M. Yang, Jian Chen, Ian Fuelscher, Jeffrey M. Craig, Timothy J. Silk

**Affiliations:** 1 School of Psychology and Centre for Social and Early Emotional Development (SEED), Deakin University, Geelong, Australia; 2 School of Medicine and the Institute for Mental and Physical Health and Clinical Translation (IMPACT), Deakin University, Geelong, Australia; 3 Neuroscience Advanced Clinical Imaging Service (NACIS), Department of Neurosurgery, Royal Children’s Hospital, Melbourne, VIC Australia; 4 Murdoch Children’s Research Institute, Melbourne, VIC Australia; 5 Department of Paediatrics, University of Melbourne, Melbourne, VIC Australia

**Keywords:** T1w/T2w ratio, MRI, Myelin, White matter, Neurodevelopment

## Abstract

Myelination of human brain white matter (WM) continues into adulthood following birth, facilitating connection within and between brain networks. In vivo MRI studies using diffusion weighted imaging (DWI) suggest microstructural properties of brain WM increase over childhood and adolescence. Although DWI metrics, such as fractional anisotropy (FA), could reflect axonal myelination, they are not specific to myelin and could also represent other elements of WM microstructure, for example, fibre architecture, axon diameter and cell swelling. Little work exists specifically examining myelin development. The T1w/T2w ratio approach offers an alternative non-invasive method of estimating brain myelin. The approach uses MRI scans that are routinely part of clinical imaging and only require short acquisition times. Using T1w/T2w ratio maps from three waves of the Neuroimaging of the Children’s Attention Project (NICAP) [*N* = 95 (208 scans); 44% female; ages 9.5–14.20 years] we aimed to investigate the developmental trajectories of brain white matter myelin in children as they enter adolescence. We also aimed to investigate whether longitudinal changes in myelination of brain WM differs between biological sex. Longitudinal regression modelling suggested non-linear increases in WM myelin brain wide. A positive parabolic, or U-shaped developmental trajectory was seen across 69 of 71 WM tracts modelled. At a corrected level, no significant effect for sex was found. These findings build on previous brain development research by suggesting that increases in brain WM microstructure from childhood to adolescence could be attributed to increases in myelin.

**Supplementary Information:**

The online version contains supplementary material available at 10.1007/s00429-023-02718-8.

## Introduction

The human brains white matter (WM) contains an abundance of myelinated axons which are critical in facilitating communication between widespread brain regions and providing trophic support to the underlying nerve fibres (Buyanova and Arsalidou 2021). The development of myelin starts in utero, rapidly progresses across the first 2–3 years of life and continues into early adulthood (Lebel and Deoni 2018). Following dendritic branching and synaptogenesis, neural connections are strengthened through axonal myelination. Magnetic resonance imaging (MRI) measures of human WM suggest it continues maturing following birth (Lebel and Deoni 2018). The period from childhood to adolescence involves dynamic brain development and reorganisation, with a crucial facet of this being axonal myelination.

Most in vivo MRI studies use diffusion weighted imaging (DWI) to examine WM development in humans. Previous work has suggested that there is an increase in microstructural properties of WM over the childhood and adolescence (Genc et al. 2018, 2020; Lebel and Beaulieu 2011; Simmonds et al. 2014; Tamnes et al. 2018). Although DWI metrics, such as fractional anisotropy (FA), could reflect axonal myelination, they are not specific to myelin and, therefore, could also represent other elements of WM microstructure, for example, fibre architecture, axon diameter and cell swelling (Beaulieu et al. 2009). Little work exists specifically examining myelin development. Imaging methods that are more specific to detect myelin, such as magnetisation transfer imaging (MTI) and myelin–water fraction (MWF), have been developed; however, these are costly and require long acquisition times, which makes utilisation for childhood cohorts difficult. Glasser and Van Essen (2011) proposed a technique using the ratio of T1-weighted (T1w) and T2-weighted (T2w) images, scans that are routinely part of clinical imaging and require short acquisition times. The technique takes advantage of the T1w relaxation period’s sensitivity to the cholesterol content in maturing myelin tissue, and T2w relaxation signal’s sensitivity to free water content in neural WM. The T1w/T2w-ratio has been validated to provide accurate estimation of neural myelination (Ganzetti et al. 2015; Glasser and Van Essen 2011), and the added ease of computation required also adds to its utility.

The T1w/T2w-ratio has been used to estimate brain myelination in healthy child (Baum et al. 2022; Norbom et al. 2020) and adult cohorts (Patel et al. 2020; Petracca et al. 2020; Uddin et al. 2019), as well as clinical adult (Baranger et al. 2021; Du et al. 2019; Luo et al. 2019; Preziosa et al. 2021)and child cohorts (Chen et al. 2022; Soun et al. 2017; Thompson et al. 2022). However, much of this work is cross-sectional, with Thompson et al. (2022)conducting the only known longitudinal modelling of brain WM myelin, examining developmental differences in preterm born children compared to those born at term. Here, they found that T1w/T2w-ratio values increase between ages 0 and 7 years for all WM tracts, excluding the left superior cerebellar peduncle and right uncinate fasciculus, with further increases for all tracts from ages 7 to 13 years.

Furthermore, the role of sex in the development of brain WM myelination is not well-understood. Longitudinal DWI and T1w/T2w ratio studies to date report conflicting results (Genc et al. 2018, 2020; Simmonds et al. 2014; Thompson et al. 2022), with either no apparent sex differences (Genc et al. 2018, 2020; Thompson et al. 2022) or increased rates of development for males in the cerebellar and limbic regions (Simmonds et al. 2014). More evidence is needed to demonstrate whether the developmental trajectories of brain WM myelin are different between males and females. This is particularly pertinent given the role of sex in neurodevelopmental disorders, such as autism spectrum disorder (ASD) and attention–deficit/hyperactivity disorder (ADHD) (for review, see May et al. 2019).

The primary aim of this study was to assess the typical developmental trajectories of myelination in major WM tracts over childhood into adolescence using the T1w/T2w-ratio technique. Second, this work aimed to assess whether developmental trajectories differed by sex. Improving on previous work, the current study adopted a non-linear longitudinal framework to assess maturational change in WM myelination over childhood into adolescence using the T1w/T2w-ratio technique. The cohort examined is the Neuroimaging in Children’s Attention Project (NICAP), a large community-based cohort consisting of typically developing children studied longitudinally across three timepoints (Silk et al. 2016).

## Materials and methods

### Participants

**Fig. 1:**
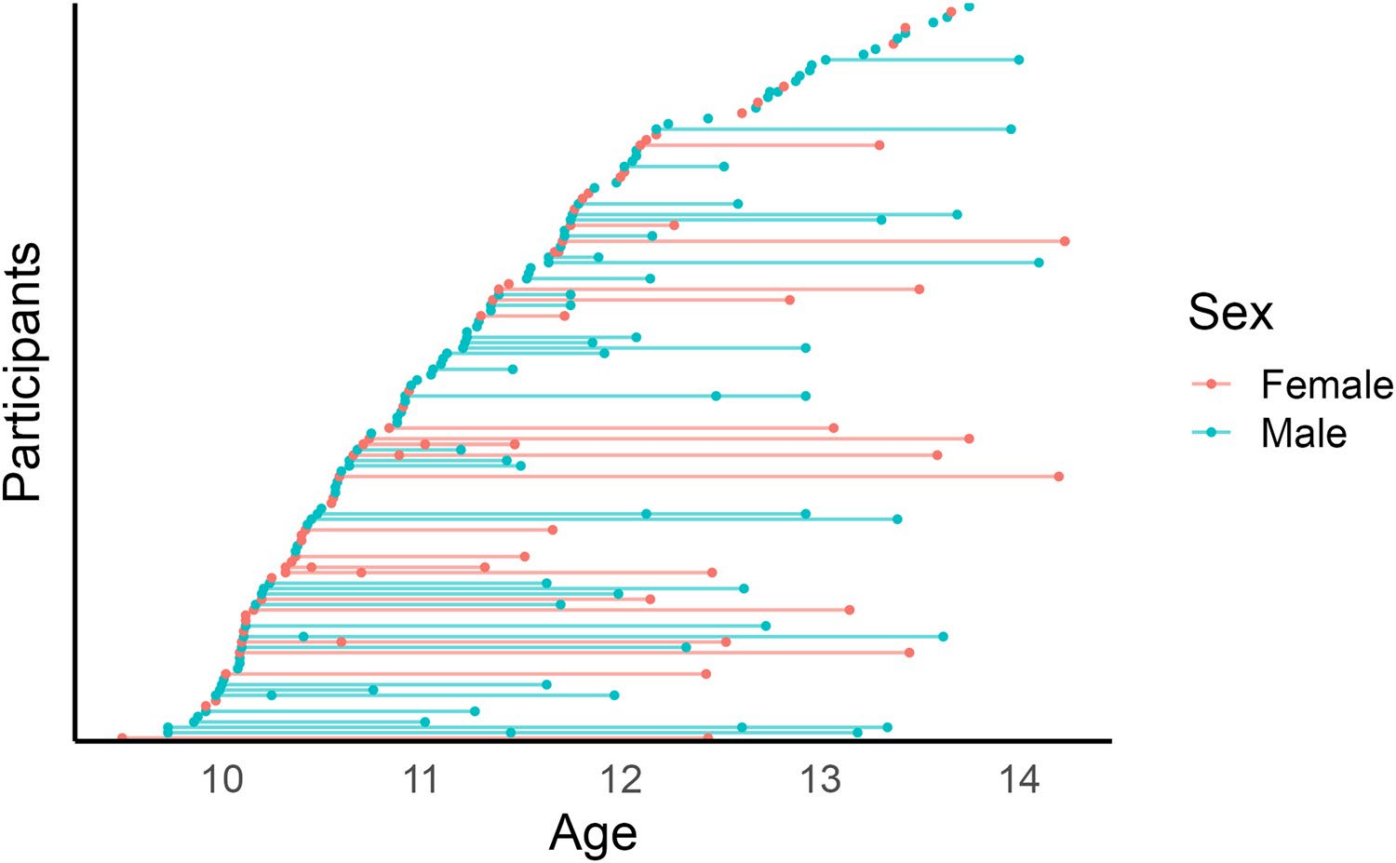
Cohort age distribution. Figure represents the distribution of participant ages across 3 points of data collection. Participants are colored by biological sex with red representing males and green representing females. Forty participants had scans at all 3 timepoints, while 34 had scans across 2 timepoints and 21 at 1 timepoint only

Data from a subsample of 95 typically developing children from NICAP (Silk et al. 2016) was used in this study. NICAP is a longitudinal single-site multimodal neuroimaging study in a community-based cohort over several years. Children were initially recruited at age 6–8 years across Melbourne, Australia, from 43 socio-economically diverse primary schools. Neuroimaging acquisition occurred at approximately the 36-, 54- and 72-month follow-up. Written informed consent was provided from all parents/guardians of the children involved in this study. The study cohort consisted of 208 scans of typically developing children acquired across three timepoints (see Table 1). Forty individuals had scans at all three timepoints, 34 at two timepoints, and 21 at one timepoint (see Fig. 1). Exclusion criteria included the presence of diagnosed neurological disorder(s), intellectual disability, or severe medical condition.

**Table 1:** Cohort demographic information

	Wave 1 (*N* = 79)	Wave 2 (*N* = 79)	Wave 3 (*N* = 50)	Overall (*N* = 208)
Age				
Mean (SD)	10.4 (0.42)	11.8 (0.49)	13.1 (0.53)	11.6 (1.15)
Range	9.50–11.9	10.9–13.4	12.2–14.2	9.50–14.2
Sex				
Male	45 (57.0%)	44 (55.7%)	29 (58.0%)	118 (56.7%)
Female	34 (43.0%)	35 (44.3%)	21 (42.0%)	90 (43.3%)

This table outlines the key demographic numbers for the cohort

### Magnetic resonance imaging (MRI) acquisition

For a comprehensive overview of the imaging protocol see Silk et al (2016). In brief, a 3T Siemens Scanner was used to conduct multimodal MRI, including structural T1-weighed (T1w), and T2-weighted (T2w) sequences as well as high-angular resolution diffusion imaging (HARDI). For acquisition sequences, see Table S9. Data were collected on a Tim Trio Scanner at waves 1 and 2, and a MAGNETOM Prisma Scanner at wave 3 following an upgrade. See supplementary material for investigation of potential effects of scanner upgrade.

### MRI processing

#### T1w/T2w-ratio pipeline

Quality control of the T1w and T2w images was first completed via visual inspection for anomalies and motion. Pre-processing steps included bias correction (Tustison et al. 2010) and brain extraction (Smith 2002). T2w images were coregistered and resampled to the T1w image space. Using Statistical Parametric Mapping (SPM) tissue segmentation, T1w images were segmented to obtain masks for WM, grey matter, and cerebrospinal fluid (CSF). Coregistered T1w and T2w images were used to create a whole brain T1w/T2w-ratio intensity map. Functional Magnetic Resonance Imaging of the Brain’s (FMRIB) Linear Image Registration Tool (FLIRT) (Jenkinson et al. 2002; Jenkinson and Smith 2001) and non-linear registration with Advanced Normalisation Tools (ANTs) (Avants et al. 2011) were then used to warp the whole brain T1w/T2w-ratio map to MNI template space. Due to the stability of CSF constitution over time, T1w/T2w-ratio values were calibrated (normalised) by the mean cerebrospinal fluid (CSF) within each wave to account for potential scanner and signal differences over time (Fonov et al. 2009, 2011).

#### Tract-based analysis

To characterize myelin levels across major WM tracts, participant DWI scans were used to generate accurate anatomical tract regions of interest (ROI) specific to this cohort.

#### DWI pipeline

DWI processing was performed in MRtrix (Tournier et al. 2019) and MRtrix3Tissue (https://3Tissue.github.io). Quality control of the raw diffusion images were completed via visual inspection for anomalies and motion. This resulted in the use of 125 scans across the 3 timepoints. Pre-processing steps included denoising (Cordero-Grande et al. 2019; Kellner et al. 2016; Veraart et al. 2016a, b; Veraart et al. 2016a, b), eddy-current, motion and susceptibility induced distortion correction (Andersson et al. 2003, 2016, 2017; Andersson and Sotiropoulos 2016; Bastiani et al. 2019; Smith et al. 2004), bias field correction (Tustison et al. 2010) and up-sampling of images to voxel size 1.5 mm^3^. Response functions for the three primary brain tissues; WM, grey matter, and CSF, were estimated (Dhollander et al. 2019) and averaged across participants to generate group-level response functions for each tissue type. Single-shell, 3-Tissue Constrained Spherical Deconvolution (SS3T-CSD) was conducted to estimate the orientation of WM fibres, producing fibre orientation distribution maps (FODs).

To create the longitudinal FOD template, 50 participant scans (25 female, 25 male) were selected across the 3 timepoints. Each participant FOD map was then registered to this longitudinal population template using linear and non-linear registration and segmented to produce discrete fixels (Smith et al. 2013).

#### Defining white mater tracts

Tractography was performed using TractSeg, a semi-automated tractography program that uses deep-learning-based algorithms and probabilistic tractography to create tractograms from HARDI data (Wasserthal et al. 2018). This resulted in delineation of 71 WM tracts across the brain (See supplementary material for definition of all tracts). WM tract ROIs were transformed into the space of the T1w/T2w-ratio brain map using the MNI152_T1_2mm template in FSL template library.

As a final step in defining the tract ROIs, to ensure tract masks were constrained to only WM voxels, each tract ROI was additionally masked using the WM tissue segmentation performed on a group T1w population template. The longitudinal T1w population template was created in MNI space (MNI152_T1_2mm) from a representative sub-sample of the cohort. This included 60 scans of different individuals (30 males and 30 female) across the three timepoints. The template was created using ANTs *command antsMultivariateTemplateConstruction2.sh.* Tissue segmentation was performed on the template using FSL’s FAST segmentation to yield a whole-brain WM mask (Zhang et al. 2001). Following binarization, the tract masks (from DWI) and the whole-brain WM mask (from T1w) were then combined, with common regions forming the final masks for all 71 WM tracts. The FSL package FLSUTILS was used to extract tract-specific average T1w/T2w-ratio values for each participant. Values were then exported for statistical analysis.

### Statistical analysis

All statistical analyses were conducted using R (version 4.1.2). Linear and non-linear mixed effects models were run using the *lme* and *lmer* functions from the nlme and lmer4 packages (Bates et al. 2014; Pinheiro et al. 2017; R Core Team 2013). A random intercept was included in all models to account for repeated observations within the sample. Separate models were run for each tract, with false-discovery rate (FDR) being used to correct for multiple comparisons (Benjamini and Hochberg 1995). Age was mean centered.

### Developmental trajectories of brain WM myelin

To estimate the developmental trajectories of WM myelination, null (A), linear (B), and quadratic (C) models were run and then compared to find the best fitting developmental model (Models A–C). A model comparison was conducted using Analysis of Variances (ANOVAs), with more complex models being chosen over simpler models when (a) models differed significantly (*p* < 0.05) (Lewis et al. 2011) and (b) fit was deemed significantly better (D Akaike information criteria (AIC) > 2).

A. $${\text{Null Model: }}Y = \beta 0$$
B. $${\text{Linear Development Model}}:Y = \beta 0 + \beta {\text{age}}$$
C. $${\text{Quadratic Development Model}}:Y = \beta 0 + \beta {\text{age}}^{2}$$
D. $${\text{Quadratic Development by Sex Model}}:Y = \beta 0 + \beta {\text{age}}^{2} + \beta {\text{sex}}$$
E. $${\text{Quadratic Development by Sex*Age Model}}:{ }Y = \beta 0 + \beta {\text{sex*age}}^{2}$$

### Sex differences in the developmental trajectories of brain WM myelin

To estimate whether developmental trajectories of WM myelination differed significantly between males and female, a main effect of sex (D) as well as the interaction between sex and age (E) were examined (Models D and E). The sex main effect model was then compared to the developmental model, and the age x sex interaction model to the sex main effect model with complex models being chosen if they satisfied the approach outlined above.

## Results

### Developmental trajectories of brain WM myelin

**Fig. 2:**
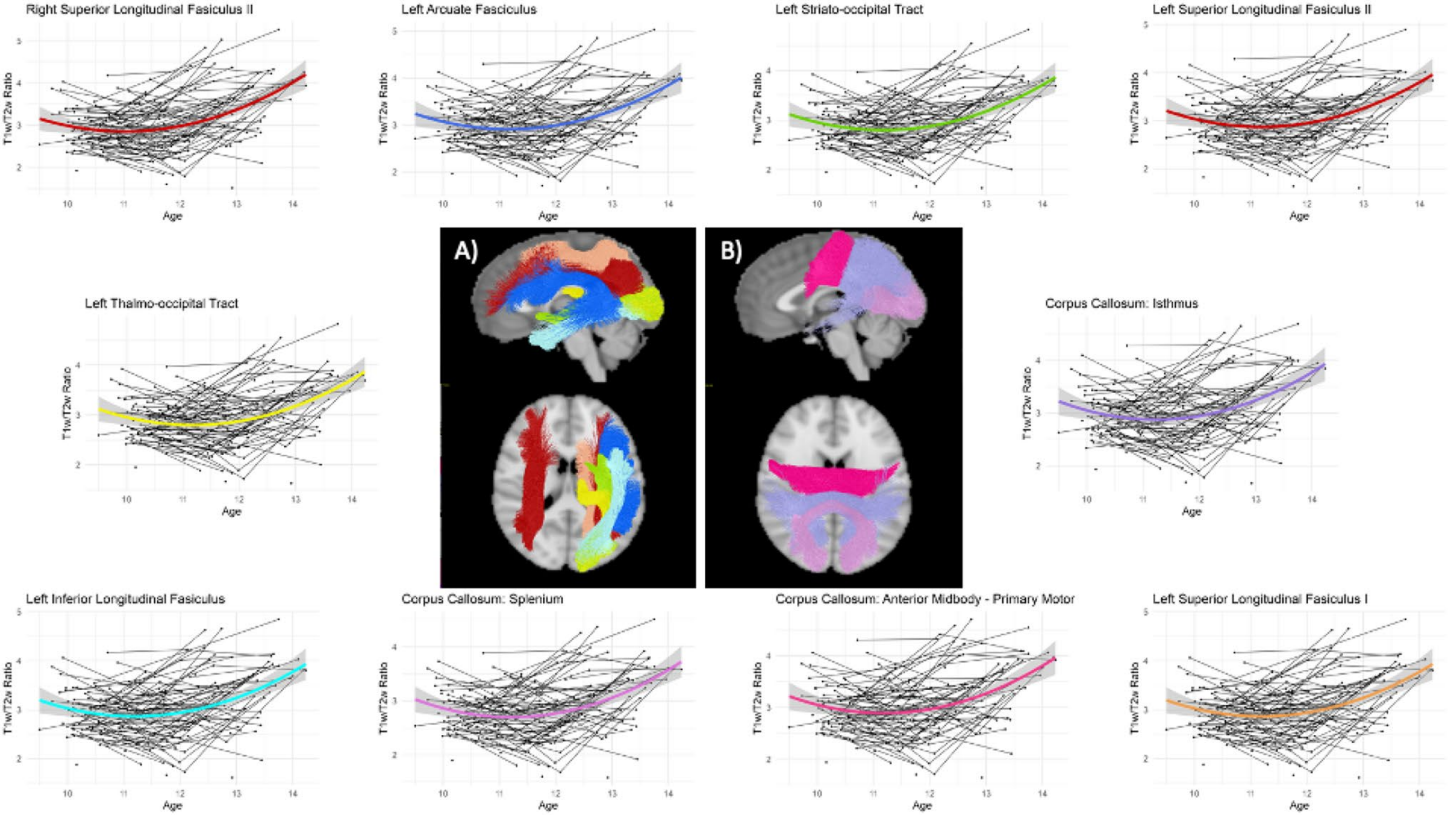
Developmental trajectories for ten most rapidly developing white Matter tracts across childhood to early adolescence. A significant effect of age was seen across all tracts. The general shape of the quadratic model was similar for most tracts, with a shallow U-shaped curve indicating more rapid development. **a** and **b** shows visual depictions of brain WM tracts with colour-matched estimated developmental trajectories of myelin

For most tracts, quadratic models provided the most parsimonious fit. For the rostrum of the corpus callosum (CC1) and the right thalamo-occipital tract (T-OCC), a linear model provided the most parsimonious fit. A significant effect of age was seen for all tracts (see Table S4). As exemplified in Fig. 2, the general shape of the quadratic model was similar for all relevant tracts, a positive shallow parabola demonstrating more rapid development in early adolescence.

The coefficient of the quadratic term was used to order the WM regions from fastest to slowest developmental progression (see Fig. S1)—excluding the CC1 and right T-OCC. This term represents the degree to which a parabolic curve is stretched or compressed. Visual representation of the top ten ranked brain WM tracts is shown in Fig. 2, see supplementary material for all modeling results.

### Sex differences in developmental trajectories

Across all tracts, neither a main effect for sex was seen (see Table S5.), nor an interaction effect (see Table S6). Like the developmental models, a positive parabolic trajectory was found to fit best, with males demonstrating more shallow trajectories over this age range; however, this difference was not significant.

## Discussion

This work builds on previous work by examining the developmental trajectories of myelin across major brain WM tracts using the T1w/T2w-ratio in a cohort of children entering adolescence. Following analysis of data across three timepoints, average T1w/T2w values increased in all WM tracts from childhood to adolescence. These developmental trajectories were best explained by a positive parabolic curve for most tracts, indicating a non-linear developmental trajectory of brain WM myelin, with more rapid increases as individuals enter early adolescence. Sex was not found to play a significant role in these developmental trajectories. The findings suggest that some of the observed changes in the brain WM microstructure from childhood to adolescence can be attributed to an increase in myelin content.

### Main effect of age

For 69 of the 71 tracts modelled, a main effect of age-squared was demonstrated. This suggests that myelination of most WM tracts follows a positive parabolic pattern (U-shaped) in relation to age, specifically between the ages of 9–15 years. For the rostrum of the corpus callosum and the right thalamo-occipital tract a linear effect of age was shown. There was little difference in rate of estimated myelination between tracts investigated. The fastest myelinating tracts included the bilateral second component (or segment) of the superior longitudinal fasciculus (SLF-II), left arcuate fasciculus (AF) and medial/posterior segments of the corpus callosum (CC) (Isthmus, Anterior Midbody, and Posterior Midbody). The right SLF-II exhibited the greatest rate of estimated myelination over this age range. Functionally, the right SLF-II is implicated in visuospatial processing (Thiebaut de Schotten et al. 2012), a cognitive process that is functional from a very early age but appears to continue developing into adolescence and early adulthood (Haist et al. 2011). Of the other top ranked tracts, the left SLF-II, AF, and isthmus of the CC are implicated in the development of language function (Brauer et al. 2011). These findings are consistent with longitudinal diffusion MRI work, whereby WM microstructure has been shown to increase over this period (Genc et al. 2018, 2020; Lebel and Beaulieu 2011; Simmonds et al. 2014; Tamnes et al. 2018). Diffusion tensor modelling across the 12–14-year age range shows similar non-linear increases in fractional anisotropy (FA), a primary measure of WM microstructure in DTI (Lebel and Beaulieu 2011; Simmonds et al. 2014; Tamnes et al. 2018). More recently, fixel-based analyses builds on previous DTI models by allowing a more nuanced investigation into brain WM microstructure, including reconciling the issue of crossing fibres (Raffelt et al. 2017). In concordance with DTI modelling and using the same data set used here (NICAP), Genc et al. (2020); Genc et al. (2018) showed that both fibre density and fibre cross section increases over two timepoints in typically developing children between the ages of 10–12 years, indicating a change in WM microstructure across a broad range of WM tracts. Our work builds on these studies by suggesting that increases in WM microstructure could be in part due to increases in axonal myelination; however, future work with more timepoints, over a larger age range, is necessary in confirming the nature of the trajectories.

### Effect of sex

To date, sex differences in the myelin development of brain WM has not been modelled in typically developing children transitioning to adolescence. Here, we used T1w/T2w ratio to specifically estimate myelination of brain WM between males and females across age. To the authors knowledge, no longitudinal T1w/T2w ratio studies exist with an age-matched cohort; however, DWI studies have shown varying results in sex differences in the development of WM microstructure (Genc et al. 2018, 2020; Lebel and Beaulieu 2011; Simmonds et al. 2014; Tamnes et al. 2018). We found no sex-related differences across any of the 71 tracts investigated. These results indicate that as children transition from childhood to adolescence, the developmental trajectories do not appear to differ between males and females. These findings align with the work of Genc et al. (2020) and Genc et al. (2018), who also did not find any significant differences in white matter microstructure between males and females of a similar, yet younger age. In contrast to our findings, however, the results of longitudinal DWI done by Simmonds et al. (2014) modelled males to have higher FA than females at ages 12–14, exhibited more protracted developmental trajectories.

Although our results here indicate no sex-related differences in the development of white matter myelin, they are not strong enough to draw robust conclusions, but instead highlight the need for future work adopt the T1w/T2w ratio to estimate neural white matter myelination from childhood, across adolescence and into adulthood.

Work here builds on previous brain development literature, suggesting that WM increases non-linearly, brain-wide from childhood into adolescence. However, results should be interpreted in light of several limitations. First, T1w/T2w values extracted were an average of all voxel T1w/T2w values contained within a tract, thus perturbations in myelination regionally within tracts could have been masked. Given appropriate computing capacity, longitudinal voxelwise modelling could provide more insight into not only fluctuations in WM myelination on a cohort level, but also allow deeper exploration of the role of sex during this important developmental period. Second, although T1w/T2w-ratio is more specific to myelin than traditional diffusion metrics, it is still only a proxy imaging marker of myelin. Resultant intensity values can thus be influenced by intra and extracellular environments, including water mobility and accumulation (Arshad et al. 2017; Ganzetti et al. 2015), as well as inflammation, hemorrhage, and infection. In this study, the T1w/T2w ratio values were scaled by the values of CSF intensities to account for differences between scans, sequences, and intensity values. This normalization method was applied as CSF intensity values are believed to be consistent over time, but this may not always be true for every individual. Considering these limitations of the T1w/T2w ratio technique, comparison with other quantitative myelin imaging methodologies, such as MWF and MTR, could help clarify our findings further.

It should also be acknowledged that, as with other longitudinal studies, our study was not impervious to changes in MRI technology. Although we took numerous approaches to ensure the scanner upgrade did not significantly influence the findings, future research should aim to replicate the findings. Similarly, numerous approaches can be taken to define white matter tracts, and how to account for longitudinal (developmental) changes at either or both the macrostructural and microstructural levels. In the current study we present one approach; however, a diversity of approaches is needed to gain a clear consensus of white-matter development. Finally, our longitudinal cohort (at wave 3) has a participation attrition rate of 24%. To account for the missing data, we applied mixed modeling, allowing participants with at least one data point to be included.

The current work builds on previous brain WM development literature in typically developing paediatric cohorts by defining WM tracts using tractography and modelling by age to detect potential non-linear trajectories. Results indicate that from childhood to adolescence, the developmental trajectory of WM myelination may increase in a non-linear fashion, not differing significantly between males and females. Further multi-modal work is necessary to confirm these results; however, this estimation of WM myelin development opens the door into investigation of WM myelination in both typical and atypical development.

### Supplementary Information

Below is the link to the electronic supplementary material.

Supplementary file1 (XLSX 135 KB)

Supplementary file2 (PDF 518 KB)

## Data Availability

Data from the Children’s Attention Project cohort are available via Lifecourse: https://lifecourse.melbournechildrens.com/cohorts/cap-and-nicap/. Code used for diffusion neuroimaging processing and analysis is publicly available and provided on the MRtrix3 (https://www.mrtrix.org) website. T1w/T2w-ratio code, as well as code used for statistical analysis can be found on github (https://github.com/ldipnall/Childhood-Development-of-Brain-White-Matter-Myelin-A-Longitudinal-T1w-T2w-ratio-Study).
