# Supplementary material for: Childhood development of brain white matter myelin: a longitudinal T1w/T2w-ratio study
Source: Brain Struct Funct. 2023 Nov 20;229(1):151–9. doi: 10.1007/s00429-023-02718-8 (PMC10827845; doi:10.1007/s00429-023-02718-8)
Supplement: Supplementary file 2 — Supplementary file2 (PDF 518 KB) [file 429_2023_2718_MOESM2_ESM.pdf]

Article Title: Childhood Development of Brain White Matter Myelin: A Longitudinal T1w/T2w-ratio Study

Document: Supplementary Material 2

Journal Name: Brain Structure and Function

Authors: Dipnall, Lillian M.[1]; Yang, Joseph YM[4,5,6]; Chen, Jian[5]; Fuelscher, Ian[1]; Craig, Jeffrey M[3,5,6]; Silk, Timothy J. [1,5]

Affiliations:
[1]Deakin University, Geelong, Australia, School of Psychology and_Centre for Social and Early Emotional Development (SEED)
[3]Deakin University, Geelong, Australia, School of Medicine and the Institute for Mental and Physical Health and Clinical Translation (IMPACT)
[4]Neuroscience Advanced Clinical Imaging Service (NACIS), Royal Children's Hospital, Department of Neurosurgery, Melbourne, VIC, Australia
[5]Murdoch Children's Research Institute, Melbourne, VIC, Australia
[6]Department of Paediatrics, University of Melbourne, Melbourne, VIC, Australia

Corresponding author: Lillian M. Dipnall; ldipnall@deakin.edu.au

Supplementary Material

Diffusion MRI Preprocessing

After initial preprocessing steps (denoising, unringing, motion correction), b0 images were extracted from main diffusion series and averaged to create a mean b0 image for each scan. T1 images were co-registered to the mean b0 image, and the co-registered T1 binarized to create an anatomical mask. Prior to bias correction (*dwibiascorrect*), a temporary diffusion mask was created using *dwi2mask*. Following *dwibiascorrect*, a new diffusion mask was created using *dwi2mask*. Due to the sensitivity of *mtnormalise* to non-brain voxels, the final diffusion mask comprised only voxels contained in both the anatomical mask and diffusion mask post-*dwibiascorrect*. All final combined masks were visually inspected to ensure unnecessary regions of non-brain tissue were not included. Final masks that did not include all brain voxels were excluded (occurred if images had incomplete coverage due to head placement or in subjects with hyperintense areas of tissue interfering with mask estimation). After upsampling to 1.5mm3 isotropic voxel size, this final combined mask was used for all future steps, including FOD estimation and *mtnormalise* (Raffelt et al., 2017).

Table S9. MRI Scanning Sequence Parameters

**Sequence**	**T1w**	**T2w**	**DWI**	
**Type**	MEMPRAGE	T2-SPACE	Shell	Blip Up/Down
**TR (ms)**	2530	3200	3200	3200
**TE (ms)**	1.77, 3.51, 5.32, 7.20	532	110	110
**TI (ms)**	1260	-	-	-
**Flip Angle (deg)**	7	-	90	90
**Slices**	176	176	63	63
**Voxel Size (mm^3)**	0.9	0.9	2.4	2.4
**FoV read (mm)**	230	240	260	260
**FoV Phase (%)**	90.6	89.8	100	100
**Matrix**	256x232	256x230	110x110	110x110
**Band Width (Hz/Px)**	723, 751, 651, 651	610	1748	1748
**Echo Spacing (ms)**	10.10	3.76	0.69	0.69

**Orientation**	S	S	T	T
**B Value (s/mm²)**	-	-	2800	0
**No. Directions/b=0**	-	-	60/4	-/2
**Multi-band factor**	-	-	3	3
**Acquisition Time**	6m 52s	4m 8s	3m 57s	35s (x2)

S = Sagittal, T = Transverse

Scanner upgrade

To investigate any effect of the scanner fit upgrade that occurred between waves 2 and 3 independent samples T-tests and Welch's T-test were conducted for all 71 tracts between age matched subgroups from waves 2 and 3 (n=20). Separate models were run for each tract, with false-discovery rate (FDR) being used to correct for multiple comparisons (Benjamini & Hochberg, 1995). The outcome variable was the T1w/T2w-ratio value for each respective tract. No significant group differences were exhibited (Table S7.).

In addition to these T-tests and Welch's T-test non-linear mixed-effect modelling of the uncalibrated data was also performed with scanner included as a covariate. Data for scanner was a dichotomous variable with 1 representing the Siemens Tim Trio Scanner at waves 1 and 2 of data collection, and 2 representing a Siemens Prisma Model and wave 3. See below for model information.

A model comparison was conducted between models A and B using ANOVAs, with more complex models being chosen over simpler models when **a)** models differed significantly (*p*<.05) (Lewis et al., 2011) and **b)** fit was deemed significantly better (D Akaike information criteria (AIC)>2).

A. $Y = \beta 0 + \beta age^2$

B. $Y = \beta 0 + \beta age^2 + Scanner$

Full results of this modelling, as well as model comparisons, can be found in Supplementary Material 2. Scanner was not found to reach significance in any of the tract models. In addition to this, model comparisons between the model with and without scanner did not yield any significant improvement in model fit.

Table S10. White Matter Tract Names

Abbreviation	Tract Name
**AF**	Arcuate fascicle
**ATR**	Anterior Thalamic Radiation
**CA**	Commissure Anterior
**CC**	Corpus Callosum – all segments
**CC 1**	Corpus Callosum: Rostrum
**CC 2**	Corpus Callosum: Genu
**CC 3**	Corpus Callosum: Rostral body (Premotor)
**CC 4**	Corpus Callosum: Anterior midbody (Primary Motor)
**CC 5**	Corpus Callosum: Posterior midbody (Primary Somatosensory)
**CC 6**	Corpus Callosum: Isthmus
**CC 7**	Corpus Callosum: Splenium
**CG**	Cingulum left
**CST**	Corticospinal tract
**MLF**	Middle longitudinal fascicle
**FPT**	Fronto-pontine tract
**FX**	Fornix
**ICP**	Inferior cerebellar peduncle
**IFO**	Inferior occipito-frontal fascicle
**ILF**	Inferior longitudinal fascicle
**MCP**	Middle cerebellar peduncle
**OR**	Optic radiation
**POPT**	Parieto-occipital pontine
**SCP**	Superior cerebellar peduncle
**SLF I**	Superior longitudinal fascicle I
**SLF II**	Superior longitudinal fascicle II
**SLF III**	Superior longitudinal fascicle III
**STR**	Superior Thalamic Radiation
**UF**	Uncinate fascicle
**T PREF**	Thalamo-prefrontal
**T PREM**	Thalamo-premotor
**T PREC**	Thalamo-precentral

**T POSTC**	Thalamo-postcentral
**T PAR**	Thalamo-parietal
**T OCC**	Thalamo-occipital
**ST FO**	Striato-fronto-orbital
**ST PREF**	Striato-prefrontal
**ST PREM**	Striato-premotor
**ST PREC**	Striato-precentral
**ST POSTC**	Striato-postcentral
**ST PAR**	Striato-parietal
**ST OCC**	Striato-occipital

Tract	Class	Age^2 Estimate with 95% CI
R Superior Longitudinal Fascicle II	Association	2.55 [1.37, 3.73]
L Arcuate Fasciculus	Association	2.29 [1.17, 3.41]
L Superior Longitudinal Fascicle II	Association	2.29 [1.15, 3.43]
CC: Isthmus	Commisural	2.27 [1.18, 3.36]
CC: Anterior Midbody	Commisural	2.25 [1.14, 3.36]
L Superior Longitudinal Fascicle I	Association	2.25 [1.15, 3.35]
L Middle Longitudinal Fascicle	Association	2.24 [1.16, 3.32]
L Striato-occipital Tract	Projection	2.23 [1.20, 3.26]
CC: Posterior Midbody	Commisural	2.22 [1.14, 3.30]
L Inferior Longitudinal Fascicle	Association	2.21 [1.14, 3.29]
L Striato-parietal Tract	Projection	2.21 [1.15, 3.28]
CC: Splenium	Commisural	2.19 [1.20, 3.18]
L Thalamo-occipital Tract	Projection	2.19 [1.17, 3.21]
R Superior Longitudinal Fascicle I	Association	2.18 [1.07, 3.30]
L Striato-precentral Tract	Projection	2.17 [1.10, 3.25]
R Superior Longitudinal Fascicle III	Association	2.16 [0.99, 3.33]
R Striato-occipital Tract	Projection	2.14 [1.13, 3.15]
R Striato-parietal Tract	Projection	2.13 [1.05, 3.21]
R Inferior Longitudinal Fascicle	Association	2.12 [1.04, 3.20]
R Optic Radiation	Projection	2.12 [1.12, 3.11]
L Striato-postcentral Tract	Projection	2.12 [1.09, 3.14]
L Thalamo-parietal Tract	Projection	2.12 [1.07, 3.17]
R Arcuate Fasciculus	Association	2.10 [0.95, 3.24]
R Middle Longitudinal Fascicle	Association	2.10 [1.01, 3.18]
L Optic Radiation	Projection	2.10 [1.08, 3.12]
L Striato-prefrontal Tract	Projection	2.10 [0.84, 3.36]
L Striato-premotor Tract	Projection	2.10 [0.97, 3.23]
R Thalamo-parietal Tract	Projection	2.10 [1.04, 3.15]
L Thalamo-precentral Tract	Projection	2.10 [1.04, 3.16]
R Striato-precentral Tract	Projection	2.09 [1.02, 3.16]
L Uncinate Fascicle	Association	2.09 [0.91, 3.27]
CC: Commissure Anterior	Commisural	2.08 [1.05, 3.11]
L Corticospinal Tract	Projection	2.06 [0.94, 3.18]
L Parieto-occipital Pontine	Projection	2.06 [0.99, 3.13]
R Thalamo-precentral Tract	Projection	2.06 [1.00, 3.12]
L Thalamo-prefrontal Tract	Projection	2.06 [0.85, 3.26]
L Inferior occipito-frontal Fascicle	Association	2.05 [0.81, 3.28]
R Superior Thalamic Radiation Tract	Projection	2.05 [0.99, 3.11]
R Cingulum	Association	2.04 [0.82, 3.26]
R Corticospinal Tract	Projection	2.04 [0.91, 3.17]
R Striato-postcentral Tract	Projection	2.04 [1.00, 3.07]
R Striato-premotor Tract	Projection	2.02 [0.88, 3.16]
CC: Genu	Commisural	2.01 [0.49, 3.53]
L Cingulum	Association	2.01 [0.79, 3.23]
L Superior Thalamic Radiation Tract	Projection	2.01 [0.95, 3.07]
L Thalamo-postcentral Tract	Projection	2.01 [1.00, 3.01]
L Thalamo-premotor Tract	Projection	2.01 [0.88, 3.14]

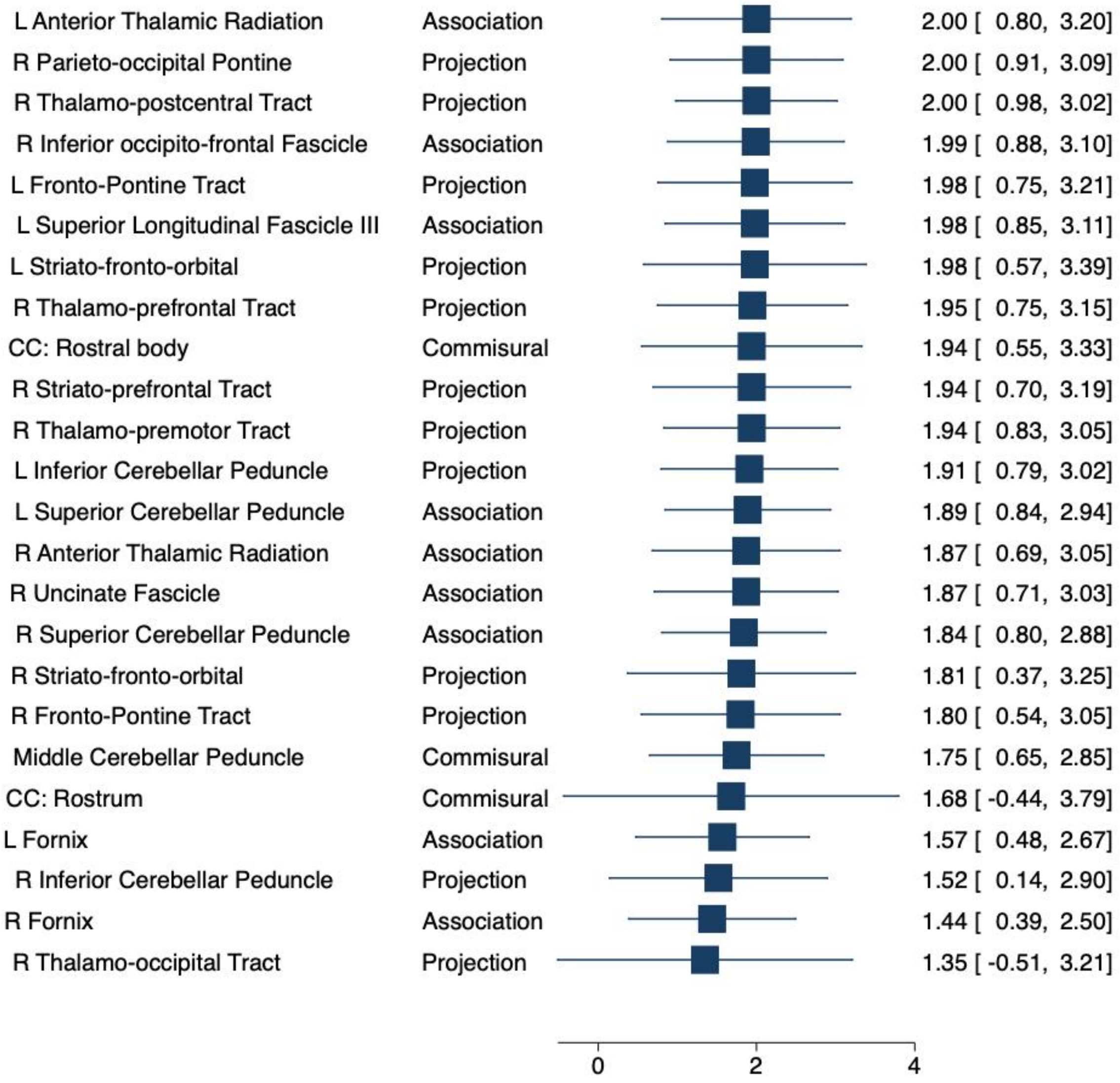

Fig. S1. Forest Plot of Brain WM Tracts Developmental Trajectories
A larger age^2 coefficient value indicates a deeper parabolic, or U-shaped curve. The navy-blue box with horizontal lines represents the age$^{2.}$ coefficient with 95% confidence intervals (CI).
R = Right; L = Left; CC = Corpus Callosum

References

Benjamini, Y., & Hochberg, Y. (1995). Controlling the false discovery rate: a practical and powerful approach to multiple testing. *Journal of the Royal statistical society: series B (Methodological)*, *57*(1), 289-300.

Raffelt, D. A., Tournier, J. D., Smith, R. E., Vaughan, D. N., Jackson, G., Ridgway, G. R., & Connelly, A. (2017). Investigating white matter fibre density and morphology using fixel-based analysis. *NeuroImage*, *144*, 58-73. https://doi.org/https://doi.org/10.1016/j.neuroimage.2016.09.029
